# Fruit quality, antioxidant, and mineral attributes of pomegranate cv. Ghojagh, influenced by shading and spray applications of potassium sulfate and sodium silicate

**DOI:** 10.1038/s41598-024-65084-3

**Published:** 2024-06-27

**Authors:** Samira Moradi, Zabihollah Zamani, Reza Fatahi, Mahmoud Koushesh Saba, Sara Paliaga, Vito Armando Laudicina, Paolo Inglese, Giorgia Liguori

**Affiliations:** 1 Department of Horticulture Science, Faculty of Agriculture, University of Tehran, Karaj, Iran; 2 Department of Horticulture Science, Faculty of Agriculture, University of Kurdistan, Sanandaj, Kurdistan Iran; 3 Department of Agricultural, Food and Forest Sciences, University of Palermo, Edificio 4, Ingresso H, 90128 Palermo, Italy

**Keywords:** Plant physiology, Plant stress responses, Biochemistry, Biogeochemistry, Climate sciences

## Abstract

Pomegranate (*Punica granatum* L.) fruit quality depends on many traits including visual, biochemical and mineral characteristics. One of the negative traits is aril whitening (AW) which is a frequently observed disorder in hot and dry climates, that leads to decline in desirable fruit quality. Color, antioxidant, and mineral contents of the arils are of prime importance as quality traits. Therefore, this study aims to investigate the effect of shading and foliar minerals on fruit quality during the fruit development stages of pomegranate. Treatments included shaded (50% green net) and unshaded trees and foliar application of trees with potassium sulfate (K, 1% and 2%) or sodium silicate (Si, 0.05, 0.1 and 0.15%) during two growing seasons. Results showed that the severity of AW at harvest decreased significantly when trees were covered with shading compared to control. The color values of L* and ⁰hue for arils were lower in fruits grown under shading conditions indicating darker red arils. Shading significantly reduced chilling injury in cold storage compared to open field fruits. Shading and Si 0.15% increased superoxide dismutase, and catalase enzymes activity while decreased Polyphenol oxidase and peroxidase. Covering trees with shading and Si 0.15% spray resulted in the highest total anthocyanin, antioxidant activity, and total phenolics content in the arils. Shading as well as Si 0.15% increased macronutrients content of the arils. The study concluded that covering pomegranate trees and spraying with Si in hot climate reduced AW, increased antioxidant traits, and led to higher fruit quality.

The occurrence of aril whitening (AW) in pomegranate (*Punica granatum* L.), also called aril paleness or aril browning by some authors, that appears mostly at hot and dry climate conditions, negatively affects the quality and market acceptance of some pomegranate cultivars^1–3^. When AW happens, the moisture content of the fruit is reduced, turning the natural red color of the arils into a light cream color. This process leads to the oxidation of polyphenols, and at severe state, the arils may turn brown^4^. Pomegranate AW begins during the growth and development of the fruit and can be observed at harvest or after storage. The external appearance of the fruit is mostly flawless, but by opening the fruit, the disorder on arils is observable. Various factors such as genotype, environmental factors (high temperature, high light intensity and low relative humidity), the internal temperature of the fruit, low water quality, reduction of Ca content and acids in the arils are responsible for the occurrence of AW^5–7^. Kavand et al.^7^ reported that the use of shading nets, increasing soil organic matter, irrigation at shorter intervals, and potassium and calcium sprays during the growing season can improve the quality of pomegranate fruits and reduce aril whitening.

In recent years, the increase in AW occurrence has been frequently reported in some commercial orchards and regions of Iran, possibly due to the adverse effects of global warming and climate change. It seems that high temperatures and high light intensity are the main factors contributing to AW during pomegranate fruit growth and development. Study has shown that the use of shade nets with different light transmittances can reduce these environmental disturbances and modify the microclimate^8^. Pomegranate cultivation in Iran is mainly centralized in areas where heat stress is frequent during the growing season. Abiotic stresses, especially high temperatures, can cause biochemical changes in plant tissues leading to oxidative damage and reduced fruit quality^9^.

Shading net and proper nutrition of plant are important strategies to reduce the effects of abiotic stresses, such as high temperature and high light intensity, on fruit trees. Shade nets make able to reduce light intensity and protect fruits from excessive sunlight as the main cause of heat stress, and improve fruit appearance^2,5^. Moradi et al.^3^ reported that high levels of bioactive compounds such as total anthocyanins, total antioxidants, phenolic compounds, and ascorbic acid, accompanied with lower aril whitening disorder in pomegranate. Increase in phenolics and antioxidant activity in pomegranate arils was reported by using shading net compared to non-shading^3,10^.

In plants, silicon (Si) is used to mitigate the effects of various abiotic stress factors such as high temperature^11^. Studies have also shown that application of Si to leaves or fruits can significantly increase the content of organic acids, phenolics, and total anthocyanins in different fruits^12–16^. Other studies have also reported the significant effect of potassium sprays on pomegranate fruit quality^17,18^. However, there is no report on the combined effect of net shading and mineral sprays on pomegranate. Present study aims to investigate the effects of Si and K spray applications combined with net shading on pomegranate aril whitening and monitoring the relationship between aril whitening and bioactive compounds at harvest and during cold storage.

## Methods

### Plant material, treatments, and storage conditions

The study was conducted in Kahak district, Qom province, Iran (34° 38′ 23.9964″ N and 50° 52′ 35.0004″ E), in two consecutive growing seasons, 2020 and 2021. Pomegranate trees cv. Ghojagh (11-year-old) were used in the study, with a tree spacing of 4 × 4 m and at North to South row orientation. The area is characterized by an arid climate with a mean annual precipitation of 132 mm per year. The region experiences hot summers with average annual temperature of 18.6 °C. In the months preceding the pomegranate harvest, the average maximum temperature of the region was 32.2 °C. The pomegranate ‘Ghojagh’ is known for its pleasant sweet–sour taste, red fruits and arils and excellent quality, being an important commodity for the regional and international markets. Treatments in this experiment consisted of net shading the pomegranate trees with 50% green cover and unshaded trees as control. In addition, mineral foliar treatments with potassium sulfate (K_2_SO_4_, Merck) at 1% and 2% concentrations (K 1% and K 2%) or sodium silicate (Na_2_SiO_3_, Merck, solution containing 25.5–26.5% SiO_2_ and 7.5–8.5% Na_2_O) at 0.05%, 0.1%, and 0.15% concentrations (Si 0.05%, Si 0.1% and Si 0.15%) were applied to the trees during the growing season. The shade nets were installed above the pomegranate trees canopy at a height of 4 m from the orchard floor in mid-June and removed two months before harvest (early September). Foliar spray applications were performed three times, once after fruit set and twice at monthly intervals (July 2, August 2, and September 2). Fruits were harvested at full maturity as determined by the experience and visual assessment of the grower for this cultivar in the region and transfered to the laboratory of the Department of Horticulture Science, Faculty of Agriculture, University of Tehran, Karaj, Iran. Fruits were stored at 5 ± 1 °C and 80 ± 5% relative humidity (RH) for a maximum of 90 days. Fruits were evaluated at the beginning, middle and end of the storage period using five fruits from each replicate (one tree as a replicate for each treatment). Factors measured on the fruit included rind and arils color, chilling damage to rind and arils, arils electrolyte leakage, total soluble solids (TSS), titratable acids (TA), and fruit weight losses. In addition, bioactive compounds such as ascorbic acid (AA), total phenolics (TP), total anthocyanins, total antioxidant activity (TAA), and activities of antioxidant enzymes including polyphenol oxidase (PPO), superoxide dismutase (SOD), peroxidase (POD), and catalase (CAT) of arils were analyzed. Also, mineral content of the arils was measured.

### Rind and aril color assay

The lightness or brightness (L*), and hue angle (⁰hue) indices (Hue angle = arctangent b*/a*) that are commonly used in color analysis of fruits and other agricultural products are helpful for determination of color characteristics and for judgment about their quality. The L index measures the brightness or darkness, ranging from 0 (black) to 100 (white). In the case of pomegranates, the L index can be used to determine how dark or light is the color of the fruit surface or arils. The hue angle index represents the dominant wavelength of light reflected by an object and determines its perceived color. It is measured in degrees on a circular scale ranging from 0 to 360.

In this experiment L^*^ and ⁰hue of fruit surface color were measured using a colorimeter (CR -400 Minolta, Japan). A total of five fruits from each experimental unit were randomly selected to perform the measurements. The measurements for each fruit surface were taken at three different points along the equator of the fruit surface and the average was used. For the arils, measurements were applied on a pool of arils inside a petri-dish at three points and averaged. This procedure was consistent with the methodology presented by McGuire^19^.

### Evaluation of aril whitening

Evaluation of the aril whitening disorder of individual fruits at harvest and during storage was performed in a visual manner using the classification procedure (1–5 rating, 1-no sign and 5 for the highest whitening) according to Moradi et al. ^3^. The results were expressed as score.

### Fruit weight loss percentage

Quantification of fruit weight loss (WL) during the storage was performed using a mathematical equation in which the final weight after storage intervals (Wt) was subtracted from the initial weight at harvest (W0) of each fruit and divided to the initial weight.

$$ {\text{Fruit weight loss }}\left( {{\text{WL}}\% } \right)\, = \,\left( {{\text{W}}0{-\!\!-}{\text{Wt}}} \right)/{\text{W}}0 \times {1}00 $$

### Fruit soluble solids content and titratable acids

Soluble solids content (SSC) was measured using an Atago digital refractometer (Brix 0–32%, Atago Japan) under standard conditions at room temperature. Quantification of titratable acids was determined by titrating fruit juice aliquots (5 ml of the juice samples from each replicate) with 0.1 N NaOH to reach a final point of pH = 8.2, and the result expressed as citric acid percentage.

### Evaluation of chilling injury (CI) index

For quantification of chilling injury (CI), 5 fruits were scored per replicate. Fruit CI symptoms were assessed individually by observable characteristics including rind browning and pitting (rind surface). To assess the intensity of CI, a five-level rating was used, so that the extent of browning on the fruit surface was categorized as 0 for no browning, 1 for less than 25% browning, 2 for browning between 25 and 50%, 3 for browning between 50 and 75%, and 4 for more than 75% browning of the fruit surface. Then, a CI index was calculated according to Sayyari et al.^69^ using the following formula:

CI index = ((0 × number of fruits at each CI class) + (1 × number of fruits at each CI class) + (2 × number of fruits at each CI class) + (3 × number of fruits at each CI class) + (4 × number of fruits at each CI class)) / 5 (total number of fruits in the treatment) × 100.

### Electrolyte leakage of rind

Determination of the rate of electrolyte leakage was performed in each replicate using six discs of peel tissue prepared with a cork borer. The weight of the peel discs was measured (ranging from 0.18 to 0.22 g) and transferred into a vial. Then, 200X of distilled water (Volume) was added to the vial. A Crison conductivity meter was used to measure the electrical conductivity (EC) of the solution. At first, the EC was measured in a preliminary test at intervals of one, two, three, and four incubation hours, while shaken continuously throughout the incubation period. According to this test the 2-h was chosen for the incubation time (with shaking) of the rind fresh samples, as the EC increments relatively stabilized after this period. After the first measurement (EC1) of fresh samples, the vials were autoclaved at 121 ℃ for 20 min and after cooling at room temperature for 24 h the total electrolytes were determined (EC2). The rate of electrolyte leakage was expressed as percentage of the total.

### Electrolyte leakage of arils

To measure electrolyte leakage of arils, 10 arils from each replicate, after a brief wash with distilled water and blotting between two layers of paper towel, were weighed (about 4 gr), then 10 times of distilled water (Volume) was added. EC was first measured on test arils after 1, 2, 3, and 4 h of incubation with constant shaking, using a Crison conductivity meter. The EC increment was declined after three hours, so three hours incubation with shaking was established for the EC1 measurements of the arils in this study. After EC1 measurement, EC2 of the arils were measured as described for the rind samples and the electrolyte leakage of arils as percentage of total was determined.

### Antioxidant enzymes assay

From the frozen arils, 1 g of sample was homogenized with 2 ml of extraction medium consisting of 100 mM phosphate buffer (pH = 7), 0.5% (v/v) Triton X-100, and 1.5% (w/v) polyvinylpyrrolidone (PVP). The resulting homogenate was centrifuged at 12,000 g for 20 min, and the supernatant was collected for enzyme assays. All steps to prepare the enzymes extracts were performed at 4 °C.

Polyphenol oxidase (PPO) activity was determined according to the method of Kahn (1975). In this assay, 100 µl of the enzyme extract was mixed with 1.4 ml of the assay solution containing 5 mM H_2_O_2_, 100 mM citrate, 200 mM phosphate buffer (pH = 5), and catechol at a final concentration of 0.05 M. Enzyme activity was measured at 420 nm for 2 min at 24 °C using a spectrophotometer (Lambda EZ 201, PerkinElmer). Enzyme activity was expressed in units mg^-^^1^protein of the homogenate^20^.

To determine peroxidase (POX), superoxide dismutase (SOD), and catalase (CAT) activity, 0.5 g of pomegranate arils were frozen in liquid nitrogen and homogenized with 2 ml of extraction buffer (100 mM phosphate buffer, pH = 7) containing 2 mM EDTA and 1% polyvinylpyrrolidone (PVP) (w/v). The homogenate was centrifuged at 12,000 g for 20 min, and the resulting supernatant was used for the enzymes assays.

POX activity was determined by measuring the guaiacol oxidation rate in the presence of H_2_O_2_ at 470 nm for 1 min^21^. The reaction mixture contained 780 µl of 50 mM phosphate buffer (pH = 7), 60 µl of 1% guaiacol, 100 µl of enzyme extract, and 60 µl of hydrogen peroxide at a final concentration of 5 mM. Enzyme activity was expressed in units mg^-1^ protein of the homogenate.

SOD activity was determined by measuring the ability of SOD to inhibit the photochemical reduction of nitroblue tetrazolium (NBT) at 560 nm after exposure to a 20 W fluorescent lamp for 30 min at room temperature^22^. The test mixture contained 1800 µl phosphate buffer (50 mM, pH = 7), 150 µl enzyme extract, 50 µl L-methionine (12 mM), 100 µl NBT (75 µM), 50 µl riboflavin (1 mM), and 50 mM NaCO_3_ (pH = 10.2). The blank mixture had the same composition but was kept in the dark. SOD Activity was expressed as units mg^-1^ protein of the homogenate.

CAT activity was determined by measuring the rate of disappearance of H_2_O_2_ according to the method of Maehly and Chance^23^. The reaction mixture contained 2600 µl of 50 mM phosphate buffer (pH = 7.4), 200 µl of 1% H_2_O_2_, and 200 µl of enzyme extract, diluted to keep the measurements within the linear range of the analysis. The decrease in H_2_O_2_ was accompanied by a decrease in absorbance at 240 nm. Enzyme activity was expressed in units mg^−1^ protein of the homogenate. Protein measurement was according to Bradford^24^ procedure.

### Total anthocyanins of arils

Anthocyanin content was determined by applying the pH differential method described by Nakamura et al.^25^. Absorbance values were determined using a spectrophotometer (Lambda EZ 201, PerkinElmer) at wavelengths of 520 nm and 700 nm in buffers with pH values of 1.0 and 4.5. Results were expressed as milligrams of cyanidin-3-glucoside equivalent per L. For this, the difference in absorbance between the buffer systems was calculated using Eq. (1) below:

1 $$ {\text{A }} = \, \left( {{\text{A52}}0 - {\text{ A7}}00{\text{ nm}}} \right){\text{ pH 1}}.0 - \, \left( {{\text{A52}}0 - {\text{ A7}}00{\text{ nm}}} \right){\text{ pH 4}}.{5} $$

Then, the concentration of total anthocyanin (mg/L) was determined using the following Eq. (2):

2 $$ {\text{Anthocyanin }}\left( {{\text{mg}}/{\text{L}}} \right) \, = \, \left[ {\left( {{\text{A }} \times {\text{ MW }} \times {\text{ DF }} \times { 1}000} \right)/{\text{ MA}}} \right] $$

In this equation, variables such as the molecular weight of cyanidin-3-glucoside (MW = 440 g/mol), the dilution factor (DF = 10), and the coefficient of molar absorptivity (MA = 26.9) were used.

### Ascorbic acid assay

Frozen arils from each replicate weighing 0.5 g were homogenized with 1.5 ml of 5% metaphosphoric acid and then centrifuged at 12,000 g for 15 min at 4 °C. The resulting supernatant was used for ascorbic acid (AA) determination^26^. To 100 µl extract volume, 500 µl of 10% metaphosphoric acid, 300 µl of citrate buffer (pH = 4.2), and 300 µl of 2,6-dichloroindophenol (DCIP) (3%) were added and then incubated for 45 min at 4 °C. Subsequently, absorbance was measured at 510 nm using a spectrophotometer (Lambda EZ 201, PerkinElmer). Results were expressed as mg AA per 100 g aril FW using the aqueous AA standards.

### Total antioxidant capacity

In order to determine the total antioxidant capacity (TAC) and total phenolics (TP), frozen arils weighing 0.5 g was subjected to extraction using a 2 ml solution of HCl-methanol-distilled water (1: 80: 19 v/v) followed by centrifugation at 12,000 g for 15 min at 4 °C. The TAC was then assessed utilizing the 2,2-diphenyl-1-picryl-hydrazyl (DPPH) radical scavenging method of Sánchez Moreno et al.^27^. Thereafter, 800 μl of the DPPH solution was combined with 200 μl of the methanolic extract and kept in the dark for a duration of 30 min. A control sample containing 200 μl of methanol mixed with 800 μl of DPPH solution was also employed. A spectrophotometer (Lambda EZ 201, PerkinElmer) was utilized to measure the absorbance at 517 nm. The total antioxidant capacity was expressed as the percentage of DPPH radical inhibition using the following equation:

$$ TAC = \frac{A\, sample - A\, control}{{A\, control}} \times 100 $$

TAC is total antioxidant capacity and A is absorbance at 517 nm.

### Total phenolics content

The content of total phenolics (TP) in the extract of the frozen arils was determined using the Folin-Ciocalteu method^28^ with some modifications. Essentially, 70 μl of the above-mentioned extract was mixed with 250 μl distilled water, 750 μl Folin-Ciocalteu reagent in a ratio of 1:10 with water, and 800 μl Na_2_CO_3_ (7.5%, w/v). The mixture was incubated for 30 min at a temperature of 30 °C. Then, the absorbance was measured at 765 nm using a spectrophotometer (Lambda EZ 201, PerkinElmer). Results were expressed as mg gallic acid equivalent (GAE) per 100 g FW using gallic acid standards.

### Mineral content analysis

The content of macro- and micro-nutrients, including Ca, Mg, K, Si, Fe, Na, Cu, Mn, and Zn, in the fruit aril samples were analyzed according to dry weight of arils. The determination was performed using MP-AES (Agilent 4210 MP-AES, Milan, Italy) after mineralization of the dry aril samples by wet digestion with HNO_3_ plus 30% H_2_O_2_. First, 0.25 g of the dried aril samples were weighed and placed in a porcelain crucible. Then, the samples were exposed to a temperature of 500 °C in a furnace for approximately 4 h, after which 5 ml of 2% HNO_3_ and 5 ml of 30% H_2_O_2_ were added. The samples were allowed to stand overnight. The digested sample was then transferred to a volumetric flask and brought to a volume of 15 mL with 2% HNO_3_^29^.

Total nitrogen content (TN) of arils was determined by the Kjeldahl method^30^. First, 0.5 g of the dried arils sample was weighed into the digestion tube, then 5 mL of concentrated sulfuric acid and a small amount of CuSO_4_, as catalyst, were added. The sample was then mineralized at 360 °C for 5 h using the DKL20 digestion apparatus (VELP® Scientifica, Italy). After cooling to room temperature, the Kjeldahl distillation apparatus UDK (VELP® Scientifica, Italy) and a Metrohm automatic titrator (916 Ti-Touch, Italy) were used to determine the TN content^30^.

For determination of total phosphorus (P), 0.5 ml of mineralized sample was mixed with 4.5 ml of H_2_O, 0.25 ml of H_2_SO_4_ (2.5 N) and 1 ml of reagent solution (ascorbic acid, ammonium molybdate, antimony potassium tartrate). The absorbance of the colored complex was measured at 882 nm using a spectrophotometer (UVmini-1240, Shimadzu Italia srl, Milan Italy). The absorbance value was then compared to a calibration curve generated using standard phosphate concentrations (2.5, 5, 10, 15 and 20 ppm). The color intensity was proportional to the phosphate concentration in the sample.

### Statistical analysis

Data were subjected to analysis of variance (two- way ANOVA) aimed at identifying sources of variance related to the application of shading and no shading, potassium sulfate and sodium silicate spray applications, and interactions between shading and spray treatments. Data were reported as means along with standard errors (SE) and represented a sample of *n* = 8. When significant differences existed at *p* ≤ 0.05, comparison of means were performed using the Tukey’s HSD test. All statistical analyzes were performed using SAS software (version 9.4).

## Results

### Aril whitening (AW)

**Figure 1:**
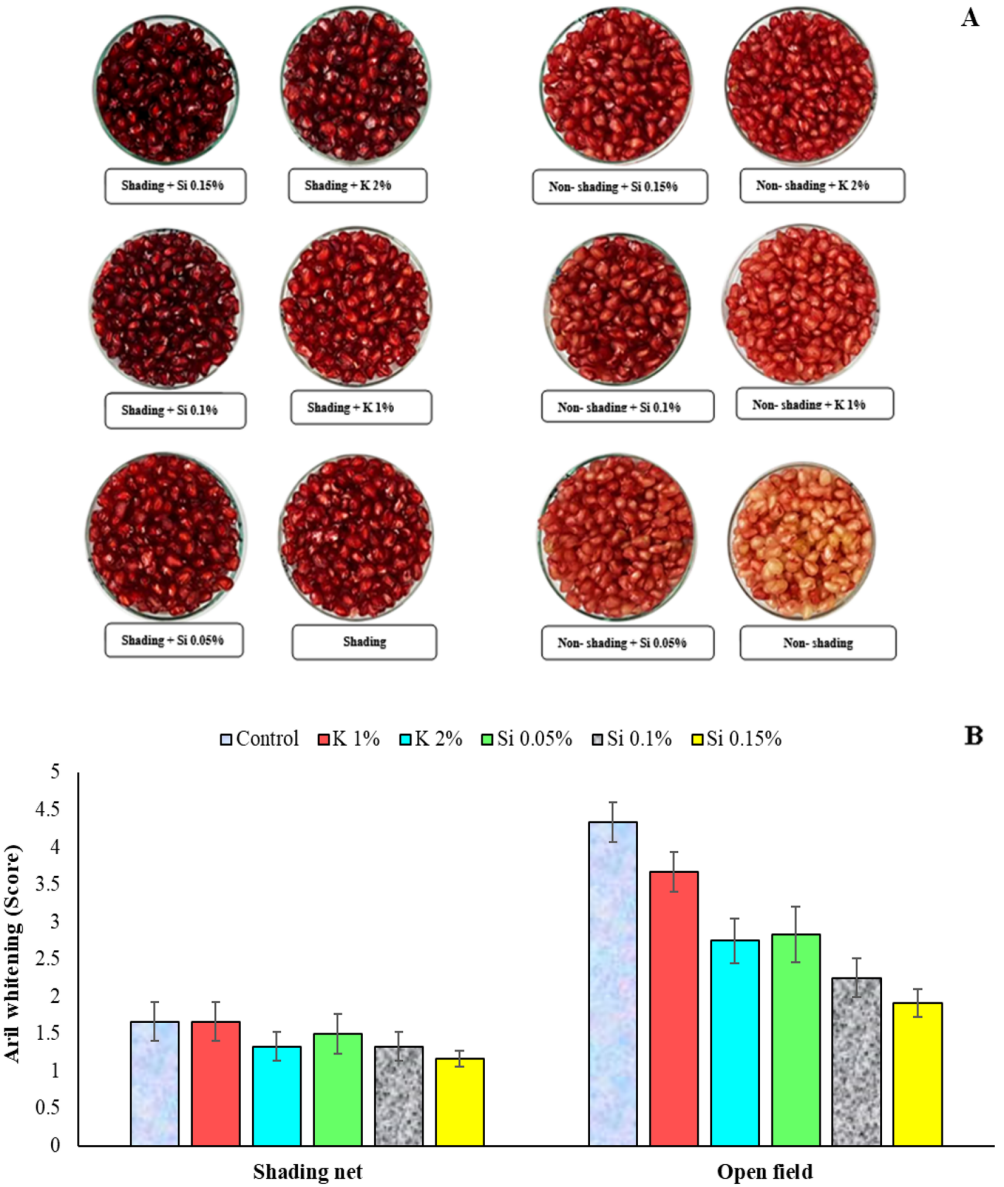
Influence of shading net and mineral spray application of Si and K on the mean of aril whitening (%) for two years (2020 and 2021) in pomegranate fruits at harvest. Means with different letters are significantly different (*p* ≤ 0.05) according to the Tukey’s HSD test (*n* = 8). Note that shading plus Si application at 0.15% or K at 2% resulted to the darker color of the arils, while no shading and no mineral treatments resulted to the arils with very pale color.

The phenomenon known as aril paleness, or AW should not be confused with natural white arils in some pomegranate cultivars, because it is basically different. White arils lack anthocyanin production from the beginning, and there are pomegranate genotypes and cultivars possessing this trait, although not much desirable for most consumers. Whereas, in AW disorder anthocyanins are synthesized in the arils during the fruit development, but whitening occurs later, accompanying with the disruption of the aril membranes, as is visible by naked eyes. This condition usually at the beginning results in paling and fading of the red color in the arils, and if progressed, results to arils with no color (white color but not transparent) and finally at the extreme condition, to browning of the arils. The arils also lose their moisture content during the process due to the damage to the membranes and become unpalatable. Results of the present study confirm the positive effect on reduction of AW using shading net and foliar spraying, both visually and statistically (Fig. 1). The use of shading net significantly reduced AW intensity compared to the open field. In addition, foliar sprays with higher concentrations of silicon and potassium showed a good effect on AW reduction, being also complementary with shading (Fig. 1A).

The effects of shade net and foliar spray treatments in present study on the occurrence of AW at harvest showed that the severity of AW was reduced from 4.33 to 1.66 in arils of pomegranate trees covered with shading net (Fig. 3B). Also, the foliar spray treatment with high silicon concentration (Si 0.15%) showed the lowest AW or the darkest aril color under the shading net (Fig. 3A and B). It was already reported that the application of 50% green shade net significantly reduced the occurrence of AW in pomegranate fruits under a hot and dry condition^3^. Similarly, Ramezani et al.^31^ also accounted that the lowest incidence of pomegranate AW was observed in fruits covered with white net. In addition, foliar application of nutrients, especially Si 0.15% was effective in reducing pomegranate AW in present study.

### Aril and rind L* and ⁰hue color attributes

**Figure 2:**
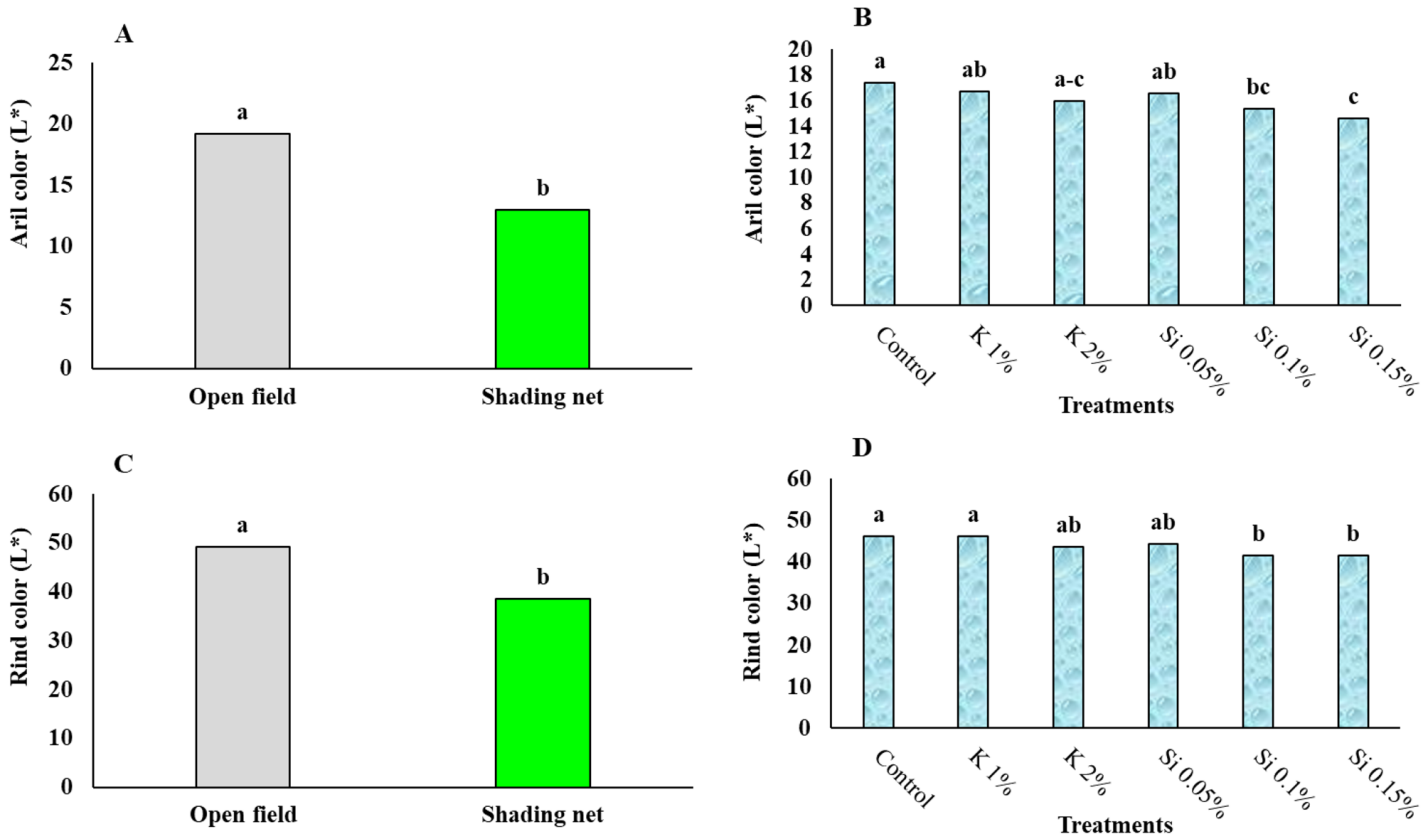
Influence of shading net and mineral application of Si and K on the mean of fruit aril and rind L^*^ color attribute for two years (2020 and 2021) in pomegranate trees during cold storage. Means with different letters are significantly different (*p* ≤ 0.05) according to the Tukey’s HSD test (*n* = 8).

**Figure 3:**
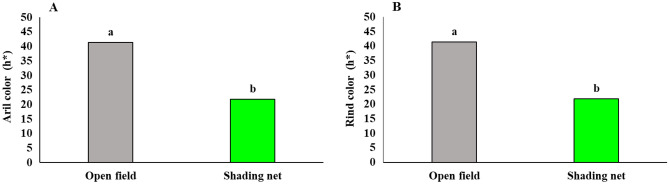
Influence of shading net on the mean of fruit aril and rind ⁰hue color attribute for two years (2020 and 2021) in pomegranate trees during cold storage. Means with different letters are significantly different (*P* ≤ 0.05) according to the Tukey’s HSD test (*n* = 8).

A higher hue angle represents less red color but a greener color, and lower L indicates a darker color of the aril and rind. Application of shading net and foliar spray treatments resulted in improved rind and aril color of pomegranate compared to those grown in open field (Figs. 2 and 3). Values of L* (aril and rind) were significantly lower in fruits grown under shading net conditions, and fruits grown under full sun showed significant higher ⁰hue values for both aril and rind (Figs. 2 A and C and 3 A and B). Si spray at higher concentrations was found to reduce L* color attributes, meaning improvement in color of both rind of pomegranate fruits (Figs. 2 B and D). Meighani et al.^1^ also reported that application of shading nets resulted in lower values of L* and ⁰hue in pomegranate fruits.

### Weight loss during storage and the chemical traits of fruit

**Figure 4:**
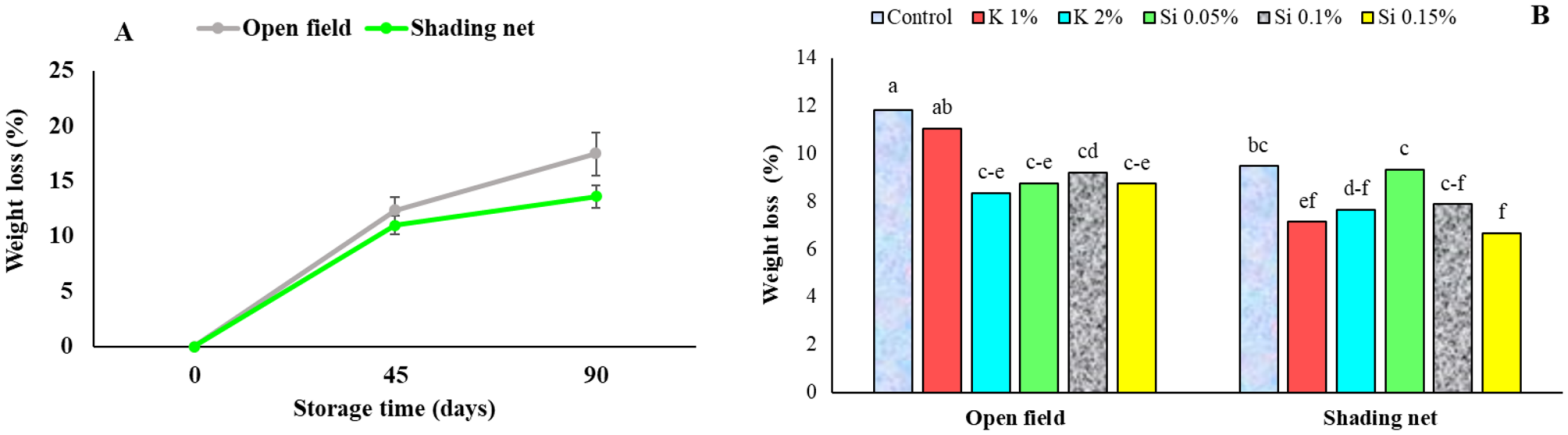
Influence of shading net (**A**) and mineral spray application of Si and K (**B**) on the mean of pomegranate fruit weight loss during cold storage (5 ± 1 °C and 80 ± 5% RH). Means (for two years of 2020 and 2021) with different letters are significantly different (*P* ≤ 0.05) according to the Tukey’s HSD test (*n* = 8).

During cold storage, a gradual increase in weight loss percentage was observed in pomegranate fruits. However, the fruits of shading net treatment showed less weight loss than the field treatment after 90 days in storage (Fig. 4A). Nutrients (Si and K) sprays also had positive effect on lowering weight loss, the Si 0.15% treatment having the lowest percent of weight loss than the other treatments (Fig. 4B). Previous reports by Tarabih^10^ and Moradi et al.^3^ represented that pomegranate grown under 35% and 50% shade nets, had significantly less weight loss during cold storage.

**Figure 5:**
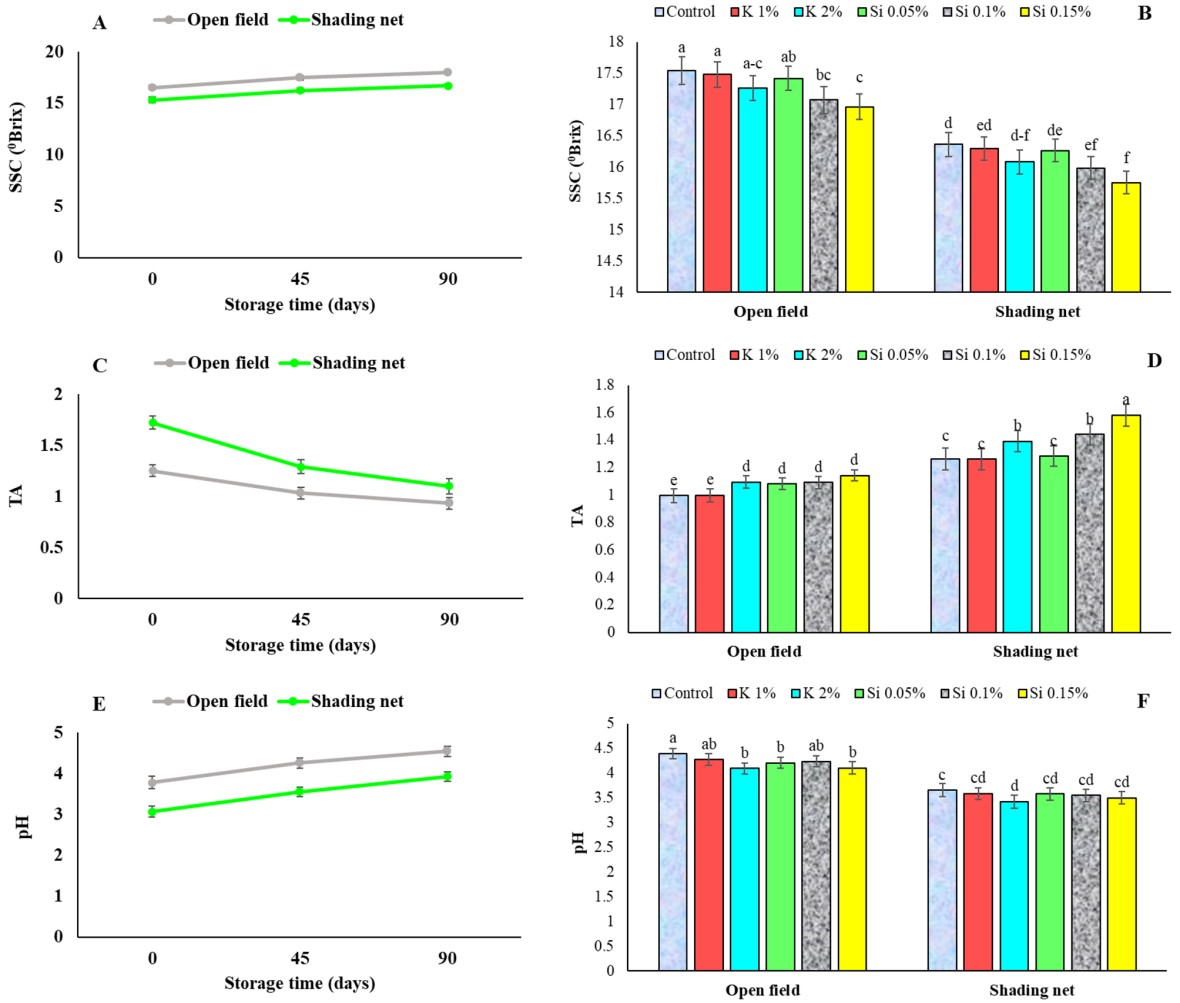
Influence of shading net and mineral application of Si and K on the mean of SSC, TA, and pH in pomegranate fruits during cold storage (5 ± 1 °C). Means (for two years, 2020 and 2021) with different letters are significantly different (*P* ≤ 0.05) according to the Tukey’s HSD test (*n* = 8).

Fruits grown under green shade nets had lower SSC, pH, and higher TA than fruits grown in open field (Fig. 5 A, C, and E). These are consistent with previous study that shade net decreased SSC content and increased TA of fruit, compared with uncovered trees^3^. In present study, foliar application of Si and K sprays, particularly 0.15% Si, resulted in a decrease in SSC and pH and increase in TA, as shown in Fig. 5B, D, and F. However, in contrast to present results, Ahmed and Gaber^16^ found that foliar application of Si decreased TA and increased TSS compared with the control. The higher TA content of the fruit under shading net in the present study is also in accordance with previous reports on pomegranate^3^ and apple^32^.

### Chilling injury in storage

**Figure 6:**
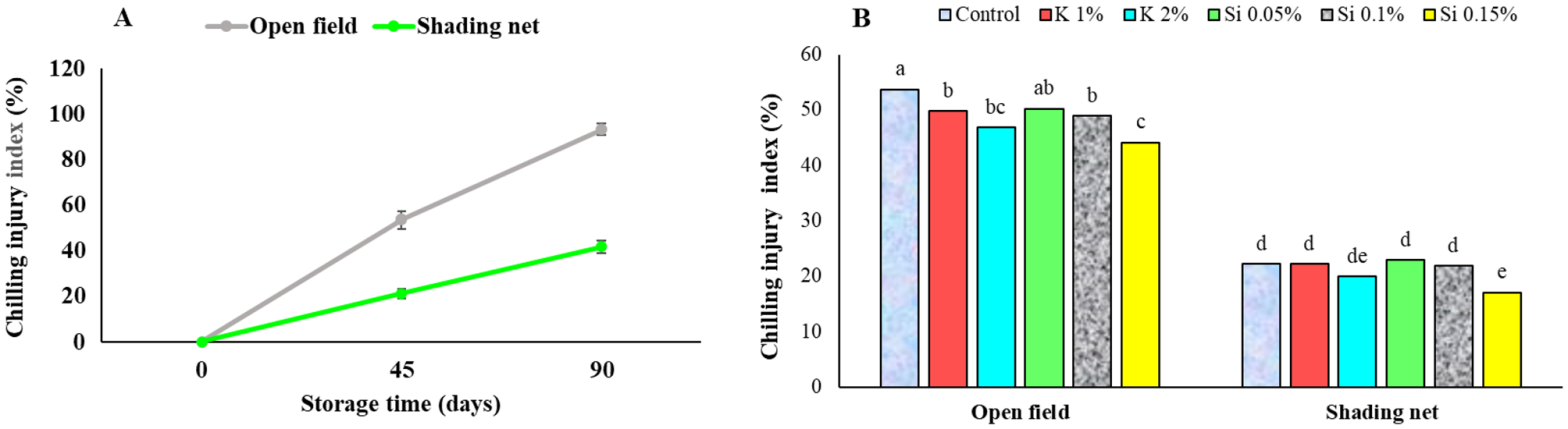
Influence of shading net and mineral application of Si and K on the mean of chilling injury index (%) in pomegranate fruits during 90 days of cold storage (5 ± 1 °C). Means (for two years, 2020 and 2021) with different letters are significantly different (*P* ≤ 0.05) according to the Tukey’s HSD test (*n* = 8).

After 90 days of cold storage, fruits from open field trees showed chilling injury symptoms more than twice than in fruits from the trees under shading net (Fig. 6A). Shading net on trees had a significant effect on reducing chilling injury of pomegranate fruits in the storage. Also, treatment with 0.15% Si was effective on reduction of the damage (Fig. 6A, B). The results of present study are consistent with previous reports^3,10^.

### Electrolyte leakage of arils and rind

**Figure 7:**
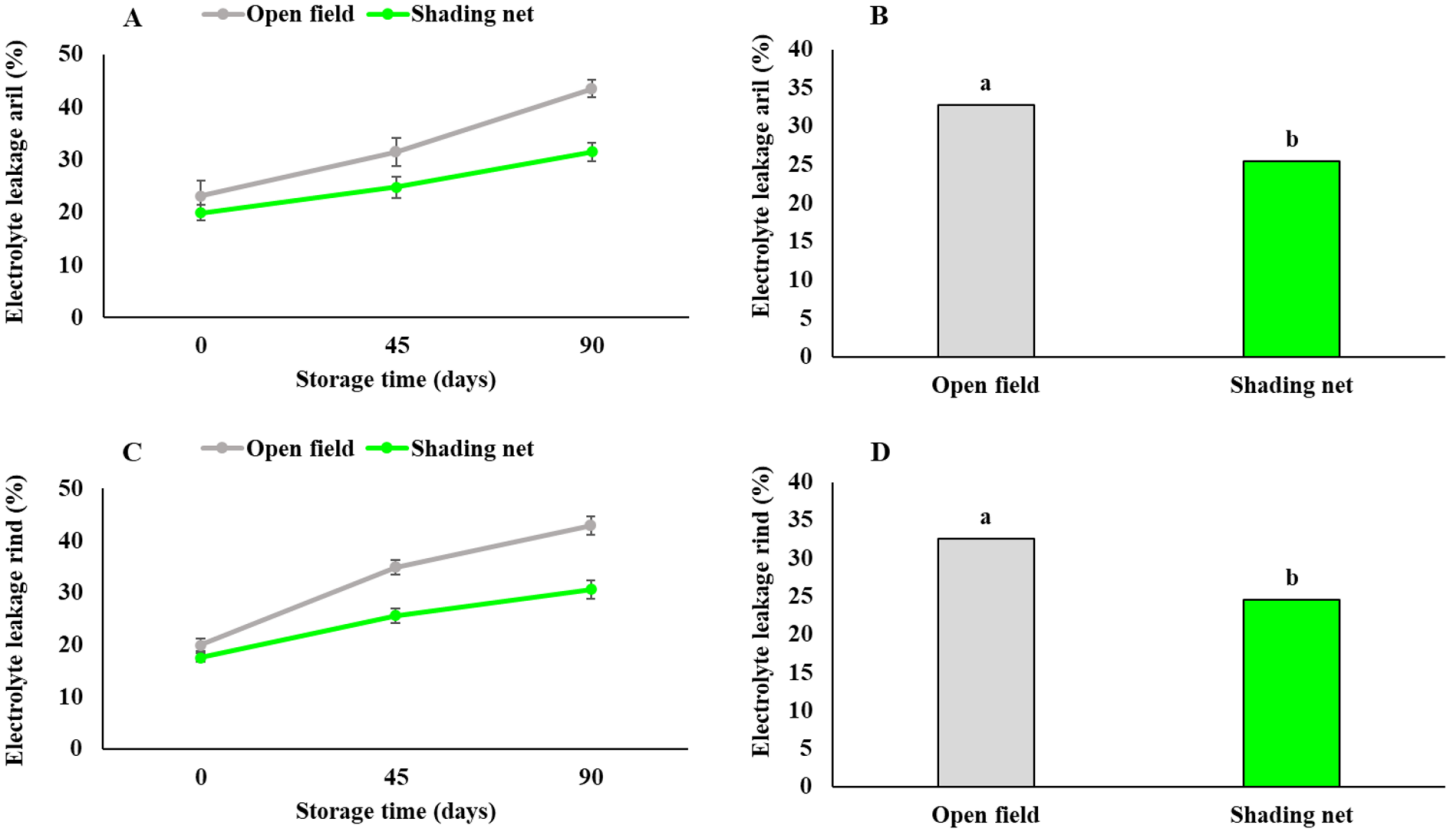
Influence of shading net on the mean of electrolyte leakage of arils and rind (%) of pomegranate fruit during cold storage (5 ± 1 °C). Means (for two years, 2020 and 2021) with different letters are significantly different (*P* ≤ 0.05) according to the Tukey’s HSD test (*n* = 8).

Shading of pomegranate trees with a green 50% net compared to non-shading trees, positively reduced electrolyte leakage (EL) from both arils and the rind of fruits during storage (Fig. 7A and C). Electrolyte leakage during 90 days of cold storage increased significantly in both arils and rind, while aril and rind of fruits from the open field had higher electrolyte leakage (Fig. 7A and C). According to this figure, shading net significantly reduced electrolyte leakage from the pomegranate arils and rind compared to the open field (24.6% and 25.4% versus 32.60% and 32.7% of EL, for arils and rind respectively) (Fig. 7B and D).

### Antioxidant enzymes activities in arils

**Figure 8:**
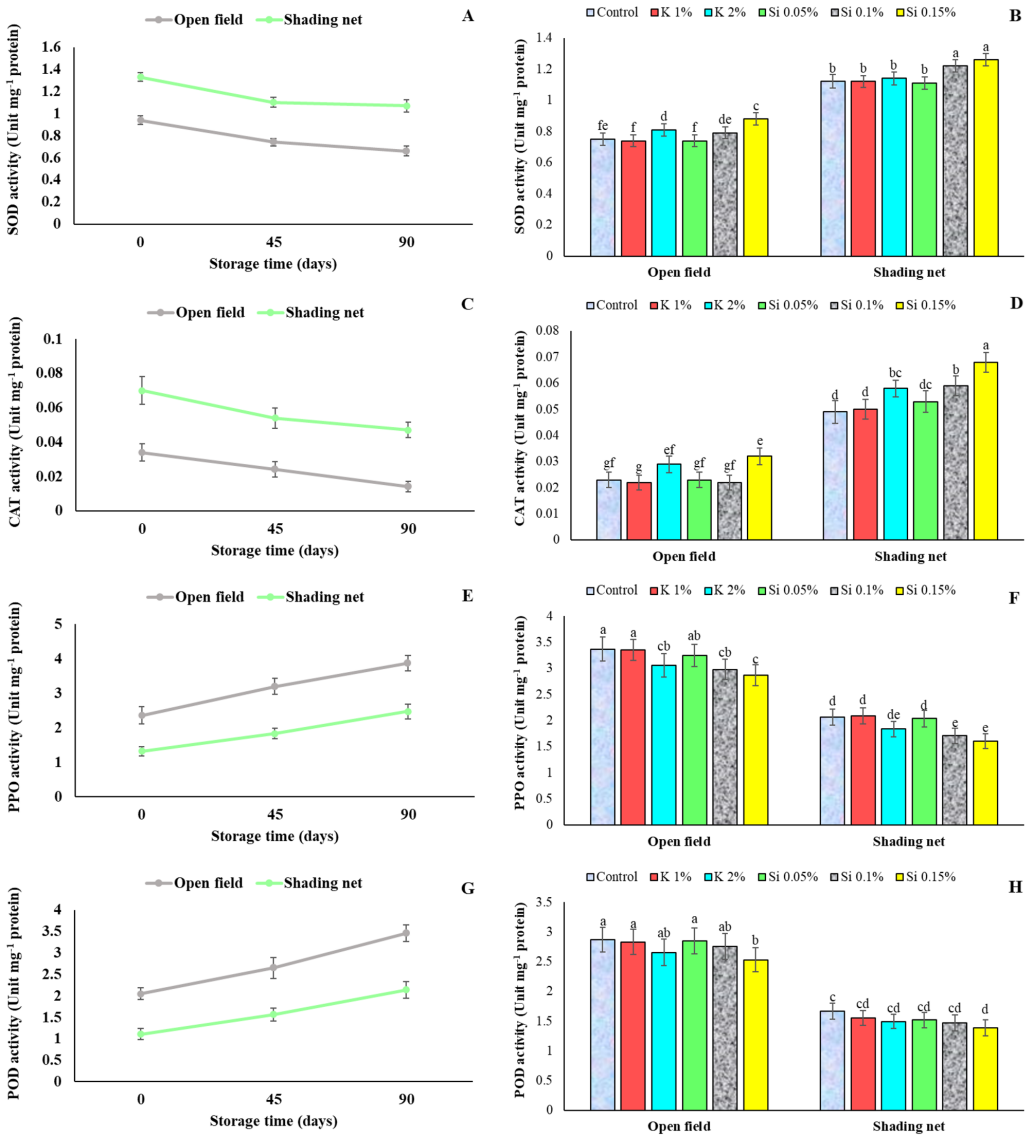
Influence of shading net and mineral application of Si and K on the mean of antioxidant enzymes activity (Unit mg^-1^ protein) in arils of pomegranate fruits during cold storage (5 ± 1 °C). Means (for two years of 2020 and 2021) with different letters are significantly different (*P* ≤ 0.05) according to the Tukey’s HSD test (*n* = 8).

Pomegranate fruit grown under 50% green shade net had significantly higher SOD and CAT enzymes activities in arils at harvest compared to those grown in open field. During the storage, the activity of SOD and CAT enzymes in the arils steadily decreased in both treatments but with keeping the higher value for shade treatment (Fig. 8A, C). The use of shading net and foliar application of silicon (specially at 0.15%) resulted in a significantly higher activity of SOD and CAT enzymes in arils of pomegranate fruit during two-year assay (Fig. 8B and D) compared with open field. It has been already reported that postharvest application of silicon increased the activity of CAT in avocado fruits^12^. In addition, research by Vieira et al.^33^ showed that potassium silicate can enhance the activity of antioxidant enzymes in mandarin seedling when exposed to biological stress. Similarly, Shi et al.^34^ reported that silicon application increased the activities of SOD and CAT enzymes in tomato seedling plant under drought stress. In addition to enzymatic antioxidants that alleviate reactive oxygen species (ROS) by silicon application, the non-enzymatic antioxidants are also important redox molecules within the cell^35^.

PPO as well as POD activities showed an increasing trend in arils of fruits from both shading and non-shading treatments during cold storage (Fig. 8E and G). Arils of fruit from trees of shading net had lower PPO and POD activity at harvest and during storage than fruits grown in full sun (Fig. 8E and G). PPO and POD activities were also influenced by mineral applications, being lowest for 0.15% Si (Fig. 8F and H).

### Total anthocyanins in arils

**Figure 9:**
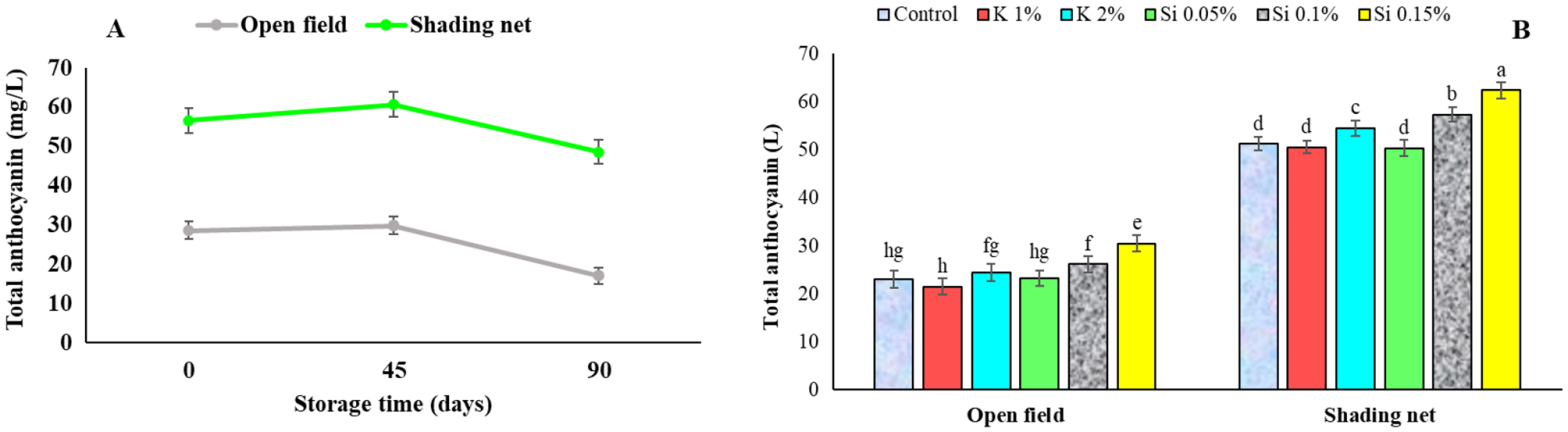
Influence of shading net and mineral application of Si and K on the mean of total anthocyanins (mg/L) of pomegranate fruits juice during cold storage (5 ± 1 °C). Means (for two years of 2020 and 2021) with different letters are significantly different (*P* ≤ 0.05) according to the Tukey’s HSD test (*n* = 8).

**Figure 10:**
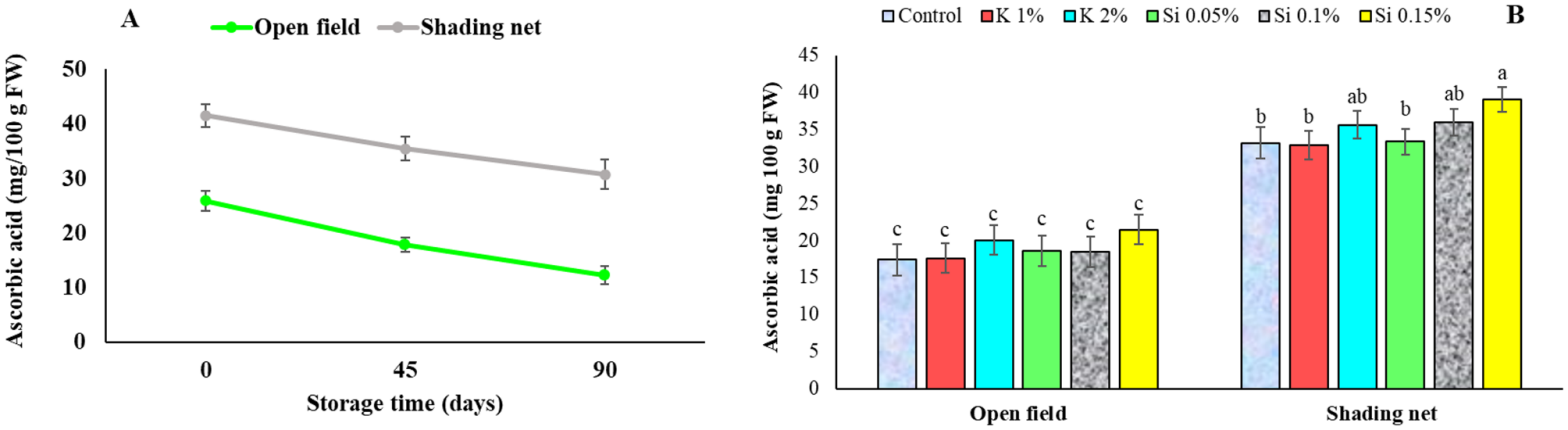
Influence of shading net and mineral application of Si and K on the mean of pomegranate fruit arils ascorbic acid content (mg/100 g FW) during cold storage (5 ± 1 °C). Means (for two years of 2020 and 2021) with different letters are significantly different (*P* ≤ 0.05) according to the Tukey’s HSD test (*n* = 8).

The use of green 50% shading net in the current study resulted in a significant increase in total anthocyanins content in the arils. The values obtained were 57 mg/100 ml of juice in the shade fruits compared to 29 mg/100 ml in the open field fruits (control trees) at harvest (Fig. 9A). In the storage, total anthocyanins content in arils showed an increasing trend until the 45th day followed by a decrease (Fig. 10A). Moradi et al.^3^ reported that green 50% shading net on trees (removed from trees 2 months before harvest), resulted to higher total anthocyanins content in pomegranate fruit rind compared to the control non-shaded trees. Foliar application of silicon, particularly Si 0.15%, resulted in a significant increase in total anthocyanins content compared with other treatments (Fig. 9B). In agreement with present results, foliar application of potassium silicate to pomegranate trees was reported to increase anthocyanin content over two growing seasons^14,16^. Also on table grape, preharvest potassium silicate application was reported to increase total anthocyanins content during two growing seasons^15^.

### Ascorbic acid

In fruits grown under shading net, ascorbic acid content of arils was significantly higher than the fruits from open field, and during 90 days storage period it decreased from 41.5 to 31 (mg/100 g FW) for shade net fruits (about 25% reduction), while it reduced from 26 to 13 (mg/100 g FW) in open field fruits (about 50% reduction) (Fig. 10A). Moradi et al.^3^ previously reported that pomegranate fruits grown under green shade net had the highest aril ascorbic acid content (40 mg/100 g FW), while those grown in the open had the lowest content (28 mg/100 g FW). The results of ascorbic acid analysis showed that Si spray treatment of 0.15% resulted in the highest ascorbic acid level for fruits of shading net compared to the other treatments (Fig. 10B).

### Total antioxidant capacity and total phenolics content of the aril

**Figure 11:**
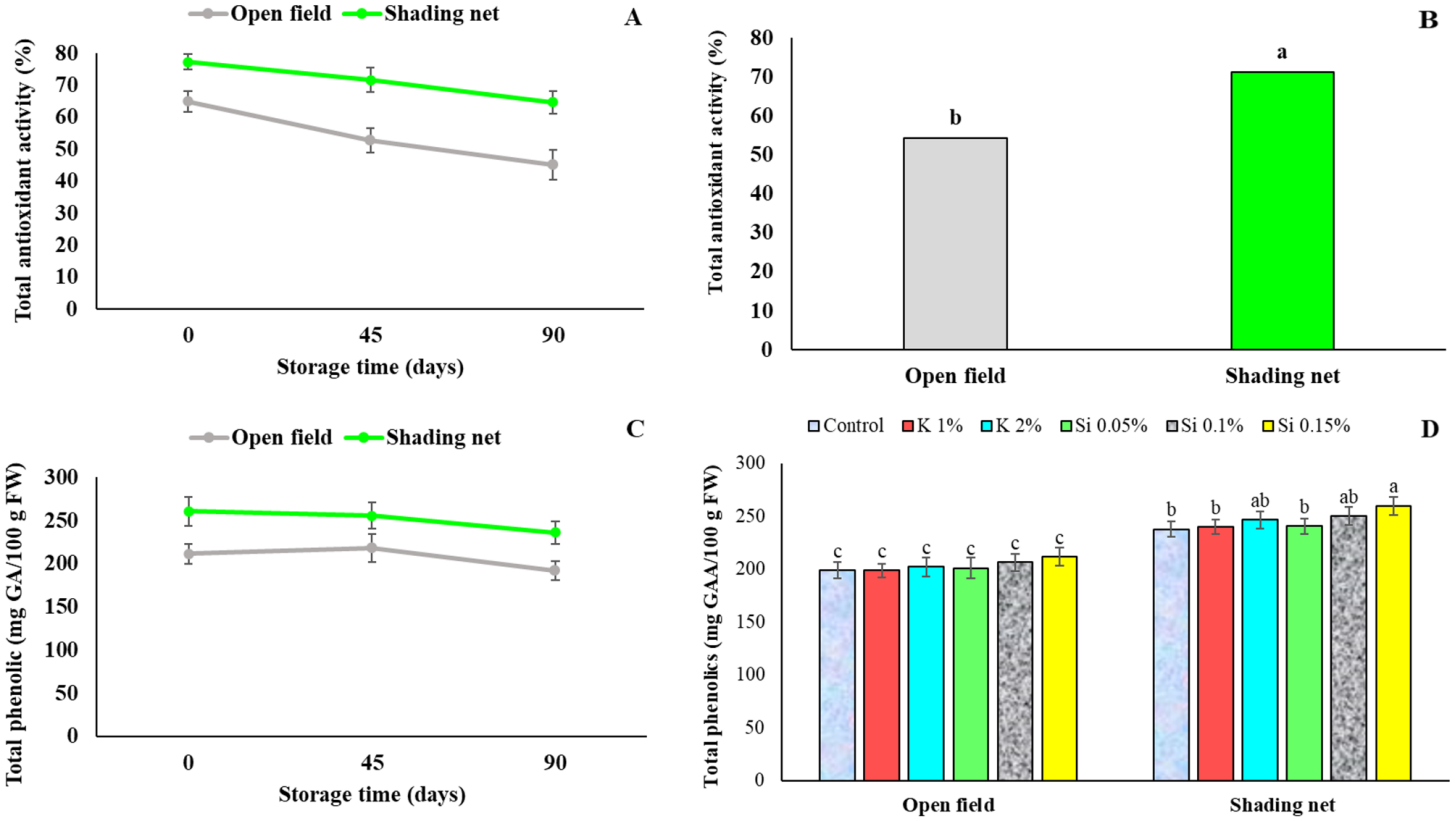
Influence of shading net and mineral application of Si and K on the mean of total antioxidant activity (%) and total phenolics (mg GAE/100 g FW) in pomegranate fruits during cold storage (5 ± 1 °C). Means (for two years, 2020 and 2021) with different letters are significantly different (*P* ≤ 0.05) according to the Tukey’s HSD test (*n* = 8).

Over the course of two years, shading net had a distinct effect on the total antioxidant activity and total phenolics content of the pomegranate arils with higher values compared to non-shading (Fig. 11). Total antioxidant activity decreased during cold storage in both shading net and open field treatments, but the amount was maintained higher in shading net treatment. (Fig. 11A). The mean antioxidant activity of arils from the shading net (71%), was higher than for the arils of fruits from open fields during two seasons (54%) (Fig. 11B). Also, fruits grown under the green shading net showed a higher total phenolics content (251 mg GA Equivalent /100 g FW at harvest), while it was lower for the open field fruits (207 mg GAE /100 g FW at harvest) (Fig. 11C). Also, a reduction of total phenolics was observed during storage period. Present results are in agreement with previous reports on the significant increase of total antioxidant activity and total phenolics content in the pomegranate fruits grown under a shade net compared to the fruits from open field^3,10,31^.

In foliar spray treatments, Si 0.15% resulted to the highest concentration of phenolics content in fruits of shaded trees (Fig. 11D). In strawberries, potassium silicate application was found to increase the phenolics content under salinity stress conditions^36^. Also, postharvest application of potassium silicate increased phenolics in lemons^37^ and avocado fruit^12^.

### Mineral nutrients

According to the present results, for fruits of trees grown under shading net, concentrations of N, P, K, Ca, and Si in pomegranate arils increased (according to dry weight of arils), while Na concentration decreased compared to fruit in full sun (Table 1). The mean of N (0.97%), P (0.64%), K (0.51%), Ca (0.123%) and Si (0.008%) according to dry weight of arils from fruits under shading net were more than the content of N (0.71%), P (0.45%), K (0.39%), Ca (0.068%) and Si (0.002%) of arils from open filed, respectively (Table 1). The shading net had no significant effect on the mineral contents of Mg, Fe, Mn, Zn, and Cu in the arils compared to non-shading (Table 1). Jokar et al.^52^ reported that N, P, Ca, and Mg concentrations in fig fruits increased by shading net, while K content was not affected. In apple fruit, using shading net increased the Ca concentration but did not affect the concentrations of Mg, K, and N^38^.

**Table 1:** Means of macro and micro elements concentrations in pomegranate arils (dry weight) as influenced by shading net and foliar spraying with Si and K. Means with different letters are significantly different (*P* ≤ 0.05) according to the Tukey’s HSD test (*n* = 8).

Treatments	Macro elements (% of DW)							Microelements (ppm/DW)			
	N	P	K	Ca	Mg	Na	Si	Fe	Mn	Zn	Cu
Non-shading	0.71b	0.45b	0.39b	0.068b	0.066a	0.054a	0.002b	22.14a	6.7a	7.15a	5.20a
Shading net	0.97a	0.64a	0.51a	0.123a	0.067a	0.046b	0.008a	23.39a	6.32a	7.57a	5.32a
HSD	0.059	0.023	0.052	0.013	0.004	0.006	0.001	3.71	0.72	1.13	0.92

Foliar treatments											
Treatments	Macro elements (% of DW)							Microelements (ppm/DW)			
	N	P	K	Ca	Mg	Na	Si	Fe	Mn	Zn	Cu
Control	0.787b	0.505b	0.426bc	0.085c	0.067b	0.056a	0.0042b	21.94a	6.54a	7.41a	4.97a
K 1%	0.785b	0.509b	0.458a-c	0.090bc	0.058c	0.049a	0.0041b	22.02a	6.39a	6.66a	5.05a
K 2%	0.818b	0.552b	0.519a	0.091bc	0.059c	0.040b	0.0046b	22.55a	6.48a	7.49a	5.07a
Si 0.05%	0.791b	0.509b	0.390c	0.085c	0.062bc	0.057a	0.0047b	22.89a	6.33a	6.66a	5.53a
Si 0.1%	0.870b	0.553b	0.444bc	0.104ab	0.075a	0.053a	0.0064a	23.33a	6.85a	7.48a	5.32a
Si 0.15%	0.999a	0.649a	0.477ab	0.119a	0.079a	0.043b	0.0067a	23.85a	6.76a	7.47a	5.60a
HSD	0.093	0.031	0.07	0.015	0.007	0.009	0.0012	3.58	1.44	1.76	1.15

*HSD* Honestly significant difference test.

In addition to shading net, spraying trees with sodium silicate (Si) and potassium sulphate (K), particularly Si 0.15%, increased the concentration of elements including N, P, Ca, Mg, as well as K and Si in the aril of pomegranate fruits. However, there is a report on cucumber plants that foliar and soil Si treatments resulted in lower K concentrations compared to the control^39^.The lowest concentrations of Na were found in the treatments with K 2% and Si 0.15%, respectively. In agreement with the results of the present study, other researchers also found that Si treatment improved the concentration of N, P, and K elements in different plants compared to the control^40–43^.

## Discussion

Aril whitening (AW) of fruit is a physiological disorder that has severely affected the quality and marketability of pomegranate fruit in recent years. When pomegranate fruits are exposed to direct sunlight at high ambient temperatures and low relative humidity, which is common in pomegranate growing areas^2^, their internal temperature can rise above 40 °C. The use of shading net on pomegranate trees significantly reduced AW intensity in fruits compared to the open field. In addition, foliar sprays with higher concentrations of silicon and potassium showed a good effect on reducing AW intensity, which also complemented with shading. The leaf and fruit temperature of pomegranate trees under shading nets decreased significantly during the growing season^6,7^. Kavand et al.^7^ reported that shading net on pomegranate treed reduced leaf, fruit and soil temperature, increased humidity and distributed sunlight in the microclimate of the studied tree. According to their results, under the shading net, the physiological processes of the trees such as photosynthesis, evapotranspiration, nutrient uptake and their translocation, as well as the synthesis of anthocyanin pigments were improved. The results reported by Ramezani et al. ^31^ show that a high anthocyanin content in the fruit is related to a lower incidence of AW. According to Kavand et al. ^2^, the stability of total phenolic compounds and anthocyanins under conditions of high TA and low PPO enzyme activity may also contribute to the reduction of AW. Present study also found that Si application increased the activity of antioxidant enzymes and bioactive compounds, which could contribute to the reduction of AW. Increase in plant tolerance to heat stress by application of exogenous Si was already reported^44^. Lowering the AW disorder intensity in present study by Si application, means increased pomegranate tolerance to harsh environment with hot temperature, which is consistent with the reported results.

The lower ⁰hue value of the arils, representing the higher red color intensity, indicated that arils had a higher content of anthocyanins under shading net as well as by application of the minerals in the present study. Higher values of L* and ⁰hue in aril and rind of pomegranate fruits grown under open fields mean lighter red color of aril and rind, and it can be an indication of the appearance of aril whitening. It should be mentioned that in the present study, the shade net was removed from the trees approximately two months before fruit harvest, when the environment temperature was reducing, which could have stimulated anthocyanin synthesis fruits upon light exposure^3^. Darker aril color represents lower aril whitening in the fruits which is approving the results of AW indexing. The enhancement of antioxidant defense systems of plants by silicon improves their tolerance potential under stress conditions^45^. Thus, it can be derived that silicon by increasing the activity of antioxidant enzymes and other bioactive compounds, to some extent reduced the adverse effects caused by heat stress, leading to the maintenance of fruit quality, including color parameters.

The main reason for weight loss in pomegranate fruit in storage is the loss of water due to the natural porosity of the rind. During the transpiration process, pomegranate fruit loses significant weight, making the arils flesh soft and less juicy and causing the rind to shrink^10^. According to Varasteh et al.^45^ the phenomenon of pomegranate fruit shrinkage gets visible when the weight decrease is more than 5%. In pomegranate fruit, the weight loss that occurs after harvest is primarily associated with the peel. Several factors can influence fruit weight loss during cold storage, including the temperature and relative humidity of the storage room, the commodity attributes, storage duration, and physiological disturbances such as cold damage. It is considered that during the hot months of the growing season, fruits grown under shade nets were less subjected to membrane damage of the tissues by high temperatures, especially in the rind, hence showing higher durability during cold storage. From the Fig. 6A, it can be anticipated that if the duration of storage was extended, the difference in weight loss percent between shading net and open field fruits would be increased.

The decrease in SSC content and increase in TA observed with silicon treatment could be due to the reduction of assimilates metabolism by alleviating stress on fruit or retarding the fruit ripening stage. Higher temperatures during tomato fruit cell division and ripening were reported to increase SSC content and was mentioned that might be related to the greater activity of carbohydrate biosynthesis enzymes and increased transpiration^47^.The higher SSC levels observed in field-grown pomegranate fruits may be due to the more photosynthetic activity of the plant under higher light intensity. Increase in SSC is also a response of plant by compatible solutes accumulation against stress conditions. Zhou et al.^47^ also reported that orange trees under the shade net had less SSC content of fruits, however the shade net increased light transmission in different directions within the covered canopy. Although shading of apple trees resulted in impaired carbon absorption, but the accumulation of secondary metabolites increased in fruits^49^. In highbush blueberries the reduction of TA was observed under colored dark nets and was accounted for lower photosynthetic activity and the reduced accumulation of carbohydrates in the fruit^50^.

Symptoms of chilling injury in stored pomegranate fruits are caused by damage to the membranes, resulting in browning of the tissues and loss of tissue integrity. Results of the current study also suggest that higher cold damage in storage on fruits grown at open field may be attributed to the increase in ion leakage as described later. It is also critical to consider the effects of changes in antioxidants and enzymes activity on reducing chilling damage as demonstrated later. These changes may be associated with membrane integrity^51^. Electrolyte leakage assay as an important indicator of cell membrane integrity, is a usual procedure for evaluating the chilling damage and other stresses. The destructive process of membrane integrity during fruit storage is resulted from the hydrolysis of phospholipids and peroxidation of membrane fatty acids, leading to accumulation of reactive oxygen species (ROS) and increased lipoxygenase activity (LOX)^52^. According to Moradi et al. ^3^, higher cell membrane integrity in fruits grown under shade-net conditions is associated with the increase in the activity of antioxidant enzymes such as CAT and SOD, leading to decrease in H_2_O_2_ concentration in the tissues. Shading net might be responsible for better maintaining membrane integrity in cells of pomegranate fruits by strengthening enzymatic and non-enzymatic defenses and diminishing free radicals from cells. In particular, higher weight loss of open field fruits (Fig. 4) is an important result of increased membrane permeability during cold storage, which is the result of cell membrane damage, which happens more or sooner in the surface tissues (rind in pomegranate) that is in straight contact with cold air^53^. Reports on higher activities of PPO and POD in browned arils of pomegranate fruits affected from heat stress, and higher SOD and CAT enzyme activities observed in pomegranate fruits under shading net are available^1,3^, and current study results are in consistent with these reports. Shivashankar et al.^4^ reported that the activity of PPO and POD enzymes increased in the damaged arils, accompanied by a decrease in total phenolics content and resulted to the browning of the arils due to enzymatic oxidation of phenolic compounds. Also, Moradi et al.^3^ reported a higher activity of PPO and POD enzymes in pomegranate fruits grown in full sun, which are subjected to aril whitening disorder, that at extreme conditions results in brown arils.

Anthocyanins are water-soluble flavonoid compounds that produce colors ranging from orange and red to various shades of blue and purple and play a crucial role in the color quality of many fresh and processed fruits. Kavand et al.^7^ reported that temperature affects the quality and quantity of anthocyanins in pomegranate fruit and their content is higher in cooler climates. A temperature of 25 °C is favorable for anthocyanin biosynthesis in grapes, but temperatures above 35 °C hinder its accumulation^54^. In the present study, the shading net was removed from the trees when the high temperatures dropped (early September), resulting to increased anthocyanin accumulation in the rind and arils, compared to the control fruit. The predominant form of anthocyanins in pomegranate fruits in warm climates is diglucoside^46^. This form is more stable but has a lower color intensity^66^. In hot and dry climates, shading nets reduce temperature and light intensity, resulting in proper PAR irradiation and increased PAL enzyme activity, which in turn causes an increase in anthocyanin content^55^. The higher anthocyanin content due to the application of shading nets as well as the use of nutrient sprays, especially the higher concentration of silicon, is consistent with the results presented in the aril whitening part of this report.

Lee and Kader^66^ reported that the most important factors that can affect ascorbic acid include: genotypic differences, cultural practices, preharvest climatic conditions, maturity level and postharvest handling procedures. The results of the ascorbic acid analysis showed that Si spray resulted in the highest ascorbic acid level for fruits of shading net compared to the other treatments. Similarly, other researchers also reported that foliar application of potassium silicate to pomegranate trees over two growing seasons resulted to a significant increase in vitamin C content of fruits compared to the control^14,16^. Ascorbic acid is an antioxidant and is sensitive to oxidative degradation leading to the formation of dehydroascorbic acid^67^. Previous studies have supported that spraying plants with potassium silicate increases the activity of antioxidant systems, thereby protecting the tissues from oxidative damage under adverse conditions^56^. According to the present study, silicon application increased the bioactive compounds including ascorbic acid, thereby significantly improved the quality of pomegranate fruit.

Phenolic compounds, including flavonoids and anthocyanins, are responsible for the antioxidant capacity of the red-blue color of various fruits^57^. Environmental factors and the microclimate in the tree canopy influence the nutritional and nutraceutical quality of the fruits^65^. In the present study, pomegranate trees under shade net resulted in higher total phenolics in the fruit arils. This might be due to the lowering of temperature and providing suitable PAR by the use of shade net in a hot and dry weather, which led to an increase in PAL activity, and consequently to the increase in phenolics content in the arils, as was also observed for anthocyanins enhancement (a cluster of phenolics) by higher PAL activity^55^. The higher content of phenolics in plants under shade nets may be related to the ability of these nets to transmit infrared and red light (an important factor for phenolics accumulation in plants) within the canopy^58^. Total phenolics in fruits from avocado trees under 20% red shade net was much higher than in fruits from trees under 20% white and 20% blue shade nets^9^. On the other hand, as well as the effects of growing and environmental conditions, phenolics content in fruits strongly depends on the plant species, pomegranate being one of the richest sources of phenolic compounds.

It is well known that the nutritional properties of pomegranate fruit are of great benefit to human, but the effects of shading net on these constituents in pomegranate have been little studied. Mobile elements such as N, P and K can rapidly transfer to fruit^59^. However, other researchers reported that shading net reduced fruit Ca content in apples and kiwifruit by up to 40%^60,61^. Shading lowers air and canopy temperature and increases relative humidity, resulting in higher water potential^54^. Hence, lower plant water potential and high evaporative demand in leaves under direct sun and high temperatures may limit Ca transfer to the fruits. Application of shading technologies have confirmed a general decrease in maximum daily temperature of 1–5 °C at some sites, followed by an increase in maximum daily relative humidity of about 3–10% under the shade^62^. Thus, shading effect on reducing the intensity of solar radiation is stronger than on air temperature. In addition to shading net, spraying trees with sodium silicate (Si) and potassium sulphate (K), particularly Si 0.15%, increased the concentration of nutrient elements including N, P, Ca, Mg, as well as K and Si in the pomegranate fruits. It has been reported that Si can change the stoichiometry of carbon (C) and phosphorus (P) in plant^42^ and increase nutrient uptake by roots^41^, leading to better N utilization. The increased uptake of Ca and K as a result of silicon application can be attributed to an increase in plasma membrane H^+^-ATP activity^63^. Zhu et al.^63^ reported that silicon application could improve cucumber plant growth under abiotic stresses by balancing nutrient uptake. Physiological and biochemical processes regulated by Si absorption in plants enhances abiotic stress tolerance by promoting or suppressing the uptake and transport of some elements in response to stress conditions.

## Conclusions

High sunlight and high temperatures in the summer months, specially at hot and arid environments, affect the quality and shelf life of pomegranate fruit. In case of heat stress alleviation, the use of shading net is recommended, as it has a positive effect on reducing temperature and light stress. In pomegranate, shading not only prevented the whitening of the arils, but also increased the quantitative and qualitative traits of pomegranate fruit, such as anthocyanins, resulting to improved color of pomegranate fruits and arils. Thus, shading net is a promising technology for reduction of physiological disorders in pomegranate, especially for alleviating of AW, and improving the fruit quality under harsh environmental conditions of high light and temperature in arid zones. Exogenous application of Si and K nutrients also had a promising effect on amelioration of the high temperature’s effects on pomegranate fruit. These nutrients, especially silicon, positively regulated antioxidant enzymes (SOD, POX, CAT) activity and improved the total anthocyanins content, as well as the antioxidant activity and phenolics content in pomegranate fruit. In general, the results show that covering the pomegranate trees in hot and dry climates for a certain period from mid-June to early September, is very effective in protecting fruits from physiological disorders such as aril whitening and enhancing its quality. Also, among the foliar spray treatments in present study, the best results are related to higher concentrations of silicon, especially Si 0.15% and also potassium 2%.

## Data Availability

The datasets analyzed during the current study are available from the corresponding author upon reasonable request.
